# Under the hood: vulvar anatomy and pathology with a focus on MRI

**DOI:** 10.1007/s00261-025-05179-1

**Published:** 2025-09-08

**Authors:** Edwarda Golden, Siobhan Cooke, Elise Butler, Lydia Gregg, Erin Gomez

**Affiliations:** 1 University of Wisconsin–Madison, Madison, United States; 2 Johns Hopkins University, Baltimore, United States

**Keywords:** Vulva, Clitoris, Labia, Vulvar malignancy, Magnetic resonance imaging

## Abstract

Vulvar anatomy and pathology can be a challenging subject to master, especially given the paucity of resources available on the subject. This review provides an overview of normal anatomy and imaging appearance of the vulva, including the mons pubis, labia majora, labia minora, clitoris, clitoral hood, external urethral meatus, vestibule and vaginal introitus, the Bartholin (greater vestibular) glands and the Skene (lesser vestibular or paraurethral) glands. We will also review the imaging appearance of various benign and malignant pathologies that affect these structures, including congenital adrenal hyperplasia, vulvar cancers, benign cysts, and urethral diverticula, with an emphasis on MR imaging. This discussion will be organized by anatomic structures to promote a thorough understanding of the pathologies which may occur in each anatomic subregion region and facilitate future referencing.

**Supplementary Information:**

The online version contains supplementary material available at 10.1007/s00261-025-05179-1.

## Introduction

The vulva and female perineum are an important but often overlooked component of women’s health. Vulvar structures play a major role in the quality of life, sexual health, and well-being of female patients. Despite its importance, myths, misinformation, and stigma have plagued both academic and societal discourse regarding female sexual health for centuries, contributing to historic disparities in the study of women’s health [1] and a paucity of information and scholarship on this topic [1, 2]. Furthermore, existing research of the female genitourinary system often focuses specifically on reproduction, overlooking other aspects of sexual health and well-being. Despite the importance of vulvar anatomy and pathology, this topic remains, for many physicians, shrouded in mystery [3]. Despite these advancements, very few resources exist which specifically focus on the imaging appearance of vulvar structures and the pathologies that can arise within them.

Over the past 25 years, there has been an increase in anatomical research and description of the clitoris and its relation to adjacent structures using cadaveric dissection and medical imaging [1, 4–6]. The widespread availability of magnetic resonance imaging (MRI) makes high resolution assessment of the vulva possible. Radiologists can now evaluate and stage vulvar malignancies, prevent unnecessary life altering surgeries, detect cancer recurrences, diagnose benign pathologies that impact quality of life, accurately describe injuries related to perineal trauma such as childbirth or female genital mutilation (FGM) (Supplemental Fig. 1), and aid in gender affirming care (GAC).

**Fig. 1:**
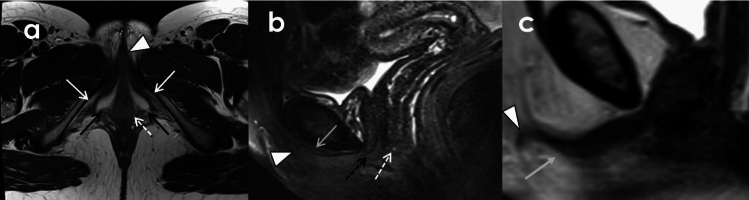
Axial T2WI (Left) and Sagittal T2WI with fat saturation (Right) demonstrating normal anatomy of the clitoris and relationship with surrounding structures in a 67-year-old female undergoing MRI for follow-up of repaired urethral diverticulum. The T2 hyperintense or intermediate vestibular bulbs of the clitoris (black arrows) flank the vaginal introitus (dashed white arrows). The T2 hyperintense crura of the clitoris run inferior to the inferior pubic rami (white arrows) and join anteriorly to form the body of the clitoris (grey arrow). The glans is ovoid in shape and intermediate in signal on T2WI (white arrow heads). The urethra (black dashed arrow) is anterior to the vaginal introitus

This review seeks to provide a foundational overview of vulvar anatomy, briefly describing relevant embryology and histology and imaging appearance of the vulva and female perineum. It will also explore many of the diseases and processes that affect the vulva and their key imaging features.

## Anatomy

The vulva is composed of the mons pubis, labia majora, labia minora, clitoris, clitoral hood, external urethral meatus, vestibule and vaginal introitus, the Bartholin (greater vestibular) glands and Skene (lesser vestibular or paraurethral) glands [7](Fig. 1).

### The mons pubis and labia majora

The mons pubis consists mainly of fat and lies superficial and cranial to the pubic symphysis. The labia majora, homologous to the scrotum in males, are twofolds of tissue composed mainly of fat and fascia (7) which envelop the labia minora and vestibule. They merge with the mons pubis anteriorly and with the perineal body posteriorly at the posterior labial commissure [7]. The mons pubis is supplied by branches of the superficial and deep external pudendal arteries and internal pudendal artery. The labia majora are supplied by the labial branches of the internal pudendal artery with some supply from the superficial and deep external pudendal arteries [7].

### The vestibule and labia minora

The vestibule is the innermost aspect of the vulva; it is bound by the clitoris anteriorly, the posterior fourchette posteriorly, and by the labia minora laterally. It contains the external urethral meatus anteriorly and the vaginal introitus posteriorly [7]. The labia minora are two smaller folds medial to the labia majora and lateral to the vestibule which extend anteriorly toward the clitoris and bifurcate. The anterior, superior bifurcations of each labium join to form the prepuce, or hood, of the clitoris. The inferior bifurcation of each labium joins to form the frenulum of the clitoris along its ventral aspect. The labia minora unite posteriorly to form the posterior fourchette [7]. The labia minora have keratinized epithelium containing sebaceous glands but without hair follicles. The transition between the keratinized epithelium of the labia minora and the nonkeratinized epithelium of the vestibule is known as Hart’s line and can be appreciated on clinical exam [7]. The labia minora are supplied by the labial branches of the internal pudendal artery. The venous drainage for both the labia majora and minora is via tributaries to the superficial pudendal vein [7].

### Clitoris

**Fig. 2:**
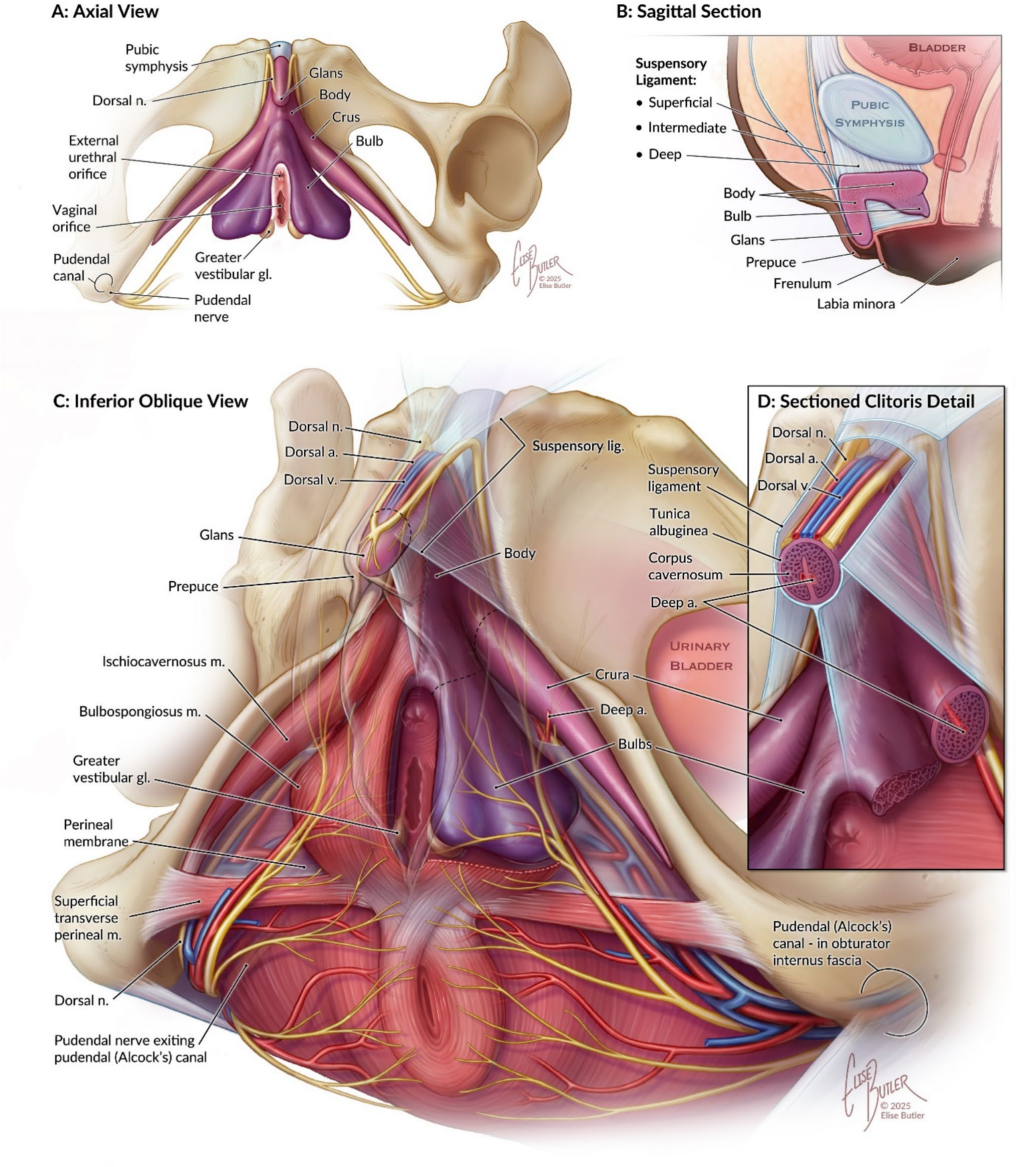
Illustrated depiction of the clitoris. **A** Inferior view, corresponding to imaging findings in the axial plane. **B** Sagittal view, corresponding to imaging findings and highlighting the clitoris, surrounding structures, and the three components of the suspensory ligament (46). **C** Inferior oblique view depicting spatial relationships of the erectile tissues of the clitoris, neurovascular supply, pelvic floor musculature, and the bony pelvis. **D** Detailed view of the clitoris, bulb of the vestibule, and associated neurovasculature in cross section. Illustration © 2025 Elise Butler

The clitoris is composed of the glans and its erectile tissue which consists of the corpus, two paired crura, and the paired bulbs of the vestibule [1] (Figs. 1, 2). The bulbs of the vestibule are paired erectile tissues that are medial to the labia minora, circling the vaginal introitus and fused anteriorly. They are covered by the bulbospongiosus muscles, which span from the perineal body posteriorly to the clitoris anteriorly. The clitoral crura are positioned laterally and attached to the inferior aspect of the pubic rami and covered by the ischiocavernosus muscles. The crura join anteriorly to form the corpus or clitoral body at the midline [1, 4]. The clitoral body is supported by the suspensory ligament, which inserts into both the public symphysis and the linea alba [1, 5, 8]. The non-suspended anterior aspect of the clitoris folds over on itself as the clitoral glans. The dorsum of the clitoral glans is covered to a varying degree by the clitoral hood or prepuce, which is formed by the anterior labia minora. The frenulum of the clitoris along the inferior aspect of the glans is formed by the anterior labia minora as well. The prepuce, frenulum and glans are the only external parts of the clitoris [1, 7].

### Bartholin glands

Bartholin glands or greater vestibular glands are small glands located within the posterolateral vagina bilaterally. They are lined by columnar epithelium and contribute to vaginal lubrication [9].

### Urethra and skene’s glands

The external urethral meatus is located in the anterior vestibule, posterior to the clitoris, and anterior to the vaginal introitus. The female urethra measures approximately 3–4 cm in length and extends from the base of the bladder to the vestibule. The distal two thirds of the urethra, including the external urethral meatus, is lined by squamous epithelium. The proximal third of the urethra is lined by urothelial cells [10]. The paraurethral (Skene’s) glands are paired mucous glands that drain into the distal urethra near the external urethral meatus [11].

### Neurovascular and lymphatic anatomy

The internal pudendal artery, which is most often a branch of the anterior division of the internal iliac artery, provides the primary vascular supply of the vulva. It serves the majority of structures inferior to the pelvic diaphragm by supplying the following arteries: the inferior rectal artery, the perineal artery, the posterior labial artery, the dorsal artery of the clitoris, the deep artery of the clitoris, and the artery of the bulb of the vestibule. The exceptions are the mons pubis and portions of the labia majora which are supplied by the external pudendal arteries arising from the femoral artery, supplied via the external iliac artery. The external pudendal arteries form anastomoses with the internal pudendal artery [7]. The venous drainage of the external genitalia is via the external and internal pudendal veins. These drain into the saphenous and internal iliac veins. Labial veins anastomose with the uterovaginal venous plexus [12]. Conditions which impact pelvic venous outflow, such as pregnancy, can result in vulvar varicosities [12].

The primary lymphatic drainage of the vulva is to the ipsilateral medial superficial inguinal lymph nodes. Structures at or close to midline can drain to either or both the left and right inguinal lymph nodes. The clitoris and anterior labia minor drain primarily to the deep inguinal lymph nodes—particularly the nodes immediately above the inguinal ring—and to a lesser extent the external iliac lymph nodes [8]. Other pelvic lymph node groups such as the pelvic sidewall and internal iliac nodes are part of the vulvar nodal basin, which has ramifications for vulvar cancer staging [13].

Innervation of the body and glans of the clitoris is provided by the dorsal nerve of the clitoris, a branch of the pudendal nerve. The labia minora receive innervation from posterior labial branches of the pudendal nerve. The labia majora are also innervated by the posterior labial nerve as well as by the anterior labial nerve arising from the genitofemoral nerve, the perineal branch of the posterior cutaneous nerve of the thigh, and the ilioinguinal nerve. The mons pubis receives innervation from branches of the ilioinguinal nerve as well [8, 14].

## Embryology

Sex differentiation begins around the seventh week of development with anatomical differences appearing by the ninth week. Initially, genital development begins with the formation of the genital tubercle at the cranial end of the cloacal membrane followed by paired labioscrotal swellings and urogenital folds which develop on either side of the cloacal membrane. In females, the genital tubercle becomes the clitoris, the urogenital folds become the labia minora, and the labioscrotal folds become the labia major. In males, the genital tubercle elongates and becomes the penis. The labioscrotal folds fuse to form the scrotum. Additional anatomic homologues include the clitoral erectile tissues, homologous to those of the penis, the paraurethral glands (Skene’s glands) which are homologues of the prostate, and the greater vestibular glans (Bartholin glands) which are homologues of the bulbourethral glands in males (Table 1) [15].

**Table 1:** Homologous anatomy

Female	Male
Labia	Scrotum
Clitoris	Glans penis
Paraurethral glands (Skene’s glands)	Prostate
Greater vestibular (Bartholin) glands	Bulbourethral glands

The internal pelvic anatomy, including the duct systems and the gonads, develops simultaneously with genital development. Initially, all embryos develop two paired ducts, the paramesonephric ducts and the mesonephric ducts which open into the abdominal cavity cranially and the urogenital sinus caudally. The paramesonephric ducts form the uterine tubes cranially, and fuse caudally to form the uterus and cervix. The vaginal canal forms when the fused paramesonephric ducts reach the urogenital sinus; this induces the outgrowth of the paired sinovaginal bulbs. Variation in the fusion of paramesonephric ducts can result in anatomical duplications (e.g., bicornate uterus). These bulbs proliferate, fuse, and form a solid vaginal plate, which becomes canalized by the fifth month of gestation. The lumen of the vagina is separated from that of the urogenital sinus by the hymen. In females, the mesonephric ducts generally regress (though see below), while in males they form duct of the epididymis, ductus deferens, and seminal gland (vesicle).

## Imaging

Many vulvar pathologies are diagnosed and monitored clinically, with a limited role for imaging. When imaging is needed, MRI is the modality of choice for nearly all pathology due to its excellent tissue resolution. CT and PET/CT can be helpful for staging locoregional nodal and distant metastatic disease, as well as detecting residual disease in treated cancers. Ultrasound and fluoroscopy can occasionally be helpful in specific clinical scenarios such as in the assessment of vascular lesions and urethral conditions.

### Imaging appearance of the vulva

**Fig. 3:**
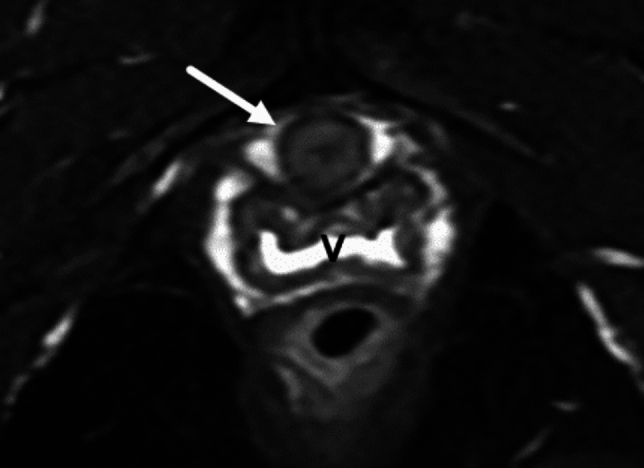
Axial T2FS of the urethra (white arrow) demonstrates a trilaminar appearance. The innermost mucosa and outer serosal layer are T2 hypointense. The inner submucosa and smooth muscle of the urethra are T2 hyperintense. *V* vagina

The labia majora is composed predominately of fat and skin and thus follows fat signal on MRI. The labia minora are thin and low in signal on T2 weighted imaging (T2WI). The clitoris including its glans, crura, and bulbs are slightly T2 hyperintense and T1 hypointense and demonstrate avid enhancement on postcontrast imaging. The muscles which cover the clitoral bulb and crura, the bulbospongiosus and ischiocavernosus, respectively are skeletal muscles and thus are T1 and T2 hypointense, however due to the fine nature of these muscles, the T2 hyperintense clitoris is almost immediately visible deep to the skin and fat. (Fig. 1). The urethra is located anterior to the vagina. It has a trilaminar, target-like appearance on T2WI and postcontrast T1 weighted imaging [16] (Fig. 3).

### Imaging protocols

MRI is the imaging modality of choice evaluating most vulvar pathology. Imaging protocols vary by indication and may need to be tailored to specific pathologies and clinical scenarios. Pelvic MRI is favored for the evaluation of vulvar cancer due to its strength in both delineating the primary tumor and evaluating nodal disease [17, 18]. The European Society of Urogenital Radiology (ESUR) recommends that protocols include T2WI, DWI-ADC, and DCE sequences [19]. Fat suppressed T2 weighted imaging is favored over non-fat saturation as it helps to delineate the primary tumor from normal perineal fat [18]. Intravenous contrast helps to visualize smaller tumors and characterize involvement of adjacent structures [18]. Diffusion weighted imaging can further aid in delineation of the tumor. Dynamic contrast enhancement is also helpful for both initial staging and distinguishing residual tumor from post-treatment fibrosis in patients undergoing therapy [19]. Patients are often advised to fast for several hours prior to MR exam. Some institutions use an antiperistaltic agent as well to minimize motion artifact from the rectum [19]. Vaginal gel can improve evaluation particularly for small vulvar tumors [20].

Protocols focused on the evaluation of the urethra require small field of view (FOV), high resolution T2-weighted images in the axial, sagittal, and coronal planes. Fat suppression can increase the conspicuity of diverticula. T1-weighted imaging is useful for identifying proteinaceous and/or hemorrhagic contents within diverticula [21]. Large FOV imaging is helpful for identifying lymphadenopathy, osseous involvement, and other metastatic disease in the case of vulvar cancers. The institutional protocol favored by the authors for evaluation of vulvar pathology is described in Table 2 and is tailored as needed to specific clinical scenarios.

**Table 2:** Recommended protocol for magnetic resonance imaging of the vulva

Technique	Pelvic phased-array coil; 1.5 or 3 Tesla
Sequences	High-resolution, thin slice (3mm), small FOV (20 cm transverse) axial and coronal T2W FS, extending from the vaginal fornices to the labia; coronal and axial sequences may be oriented parallel and perpendicular to the urethra, respectively
	Small FOV, axial, DWI/ADC*: extending from the vaginal fornices to the labia
	Small FOV, axial, pre- and postcontrast T1WI: extending from the vaginal fornices to the labia
	Large FOV, axial, T1W: pelvis
	Large FOV, axial, T2 W: pelvis

*FOV* field of view, *T2W* T2-weighted, *FS* fat saturated, *T1W* T1-weighted, *DWI* diffusion weighted imaging, *ADC* apparent diffusion coefficient. For diffusion-weighted images, b-values of 50 and 800 are recommended to generate adequate ADC maps

## Congenital conditions of the vulva

Congenital conditions affecting the vulva can be variant normal anatomy or conditions caused by atypical hormone levels or signaling changes in utero.

### Unilateral fibrous hyperplasia of the labium majus

Unilateral fibrous hyperplasia of the labium majus is unilateral, painless and nontender enlargement of the labia majus in prepubertal and early pubertal girls. Reported imaging findings include unilateral enlargement of the labia majus. The tissue of the enlarged majus may have heterogeneous echotexture on ultrasound and demonstrate ill-defined T1 hypointense, T2 hyperintense signal on MR. There are no underlying masses causing the enlargement of the labium. This entity is rare but considered benign. Recognition of this entity could prevent unnecessary treatment [22, 23].

### Congenital adrenal hyperplasia

**Fig. 4:**
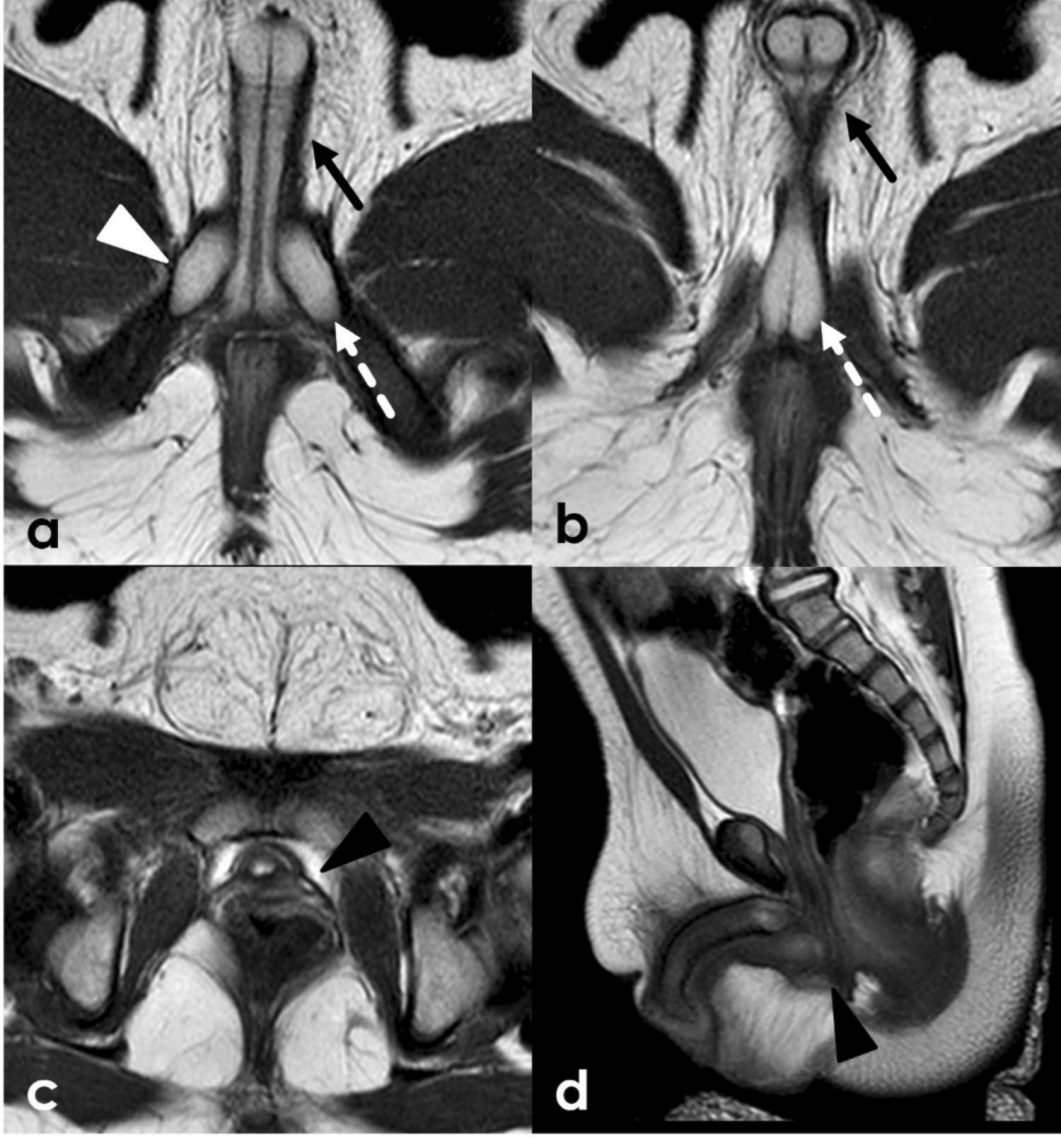
9-month-old female with congenital adrenal hyperplasia**.** Axial (**a**–**c**) and sagittal (**d**) T2WI demonstrates ambiguous genitalia with clitoromegaly (black arrows) as well as enlargement of the bulbs (white dashed arrows) and crura (white arrowhead). This patient demonstrated Prader IV anatomy with complete fusion of the labia majora and a common channel urogenital sinus (black arrowhead)

Congenital adrenal hyperplasia (CAH) is a group of congenital disorders characterized by deficiencies in different enzymes involved in adrenocorticoid steroid production. In addition to other hormone abnormalities, CAH is associated with high androgen levels. Increased androgen levels may result in virilization and ambiguous genitalia in 46, XX patients. Two major subtypes of CAH exist: classic, which presents at birth, and non-classic, which presents later in life. Despite having typical paramesonephric (Müllerian) duct development, 46, XX patients with CAH can have external genitalia that phenotypically resemble a male phallus and scrotum, though without testes. The Prader scale, developed specifically for XX infants affected by CAH, systematically uses features such as clitorophallic length and degree of labioscrotal fold fusion to categorize the degree of severity. Lower stages on the Prader scale are characterized primarily by increased clitorophallic length with labioscrotal fusion at the higher levels. At the highest stages, patients can have a common channel urogenital sinus for both the urethra and vagina [24] (Fig. 4).

### Gartner duct cysts

**Fig. 5:**
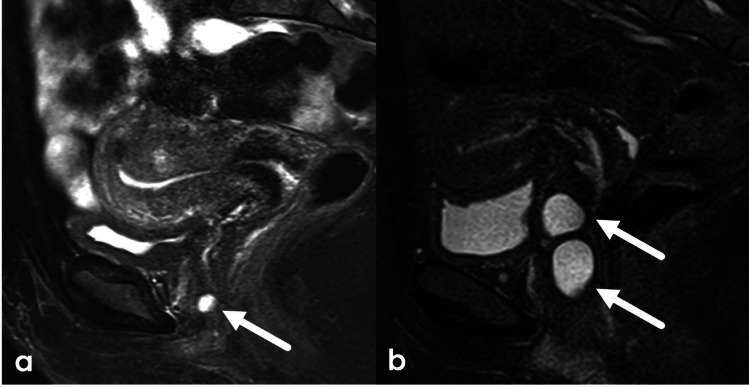
Sagittal T2WI with fat saturation demonstrating Gartner duct cysts in two separate patients: 37-year-old undergoing MRI of the pelvis for abnormal uterine bleeding (**a**), Gartner duct cyst incidentally noted, and (**b**) 29-year-old female with vaginal mass noted on annual pelvic exam

The mesonephric (Wolffian) mesonephric ducts, the precursors of the epididymis, vas deferens and seminal vesicles, are present in both males and females in utero. The absence of testosterone as produced by the male gonads allows the Wolffian ducts to regress in female patients. Ductal remnants due to failed regression in female patients are called Gartner ducts [15]. These are located in the broad ligament of the uterus and in the vaginal wall. Benign cysts can develop within the Gartner ducts [15]. On MRI, Gartner duct cysts will appear as T2 hyperintense cystic structures in the vaginal wall, often above the level of the symphysis pubis. Complex cyst components may result in varied signal characteristics on T1 and T2. Small Gartner duct cysts in the anteroinferior vaginal wall near the urethra may be difficult to distinguish from Skene’s gland cysts (Fig. 5).

## Imaging and pathology of the clitoris

### Clitoromegaly

**Fig. 6:**
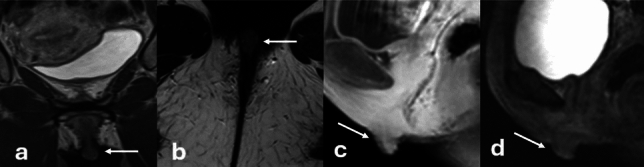
48-year-old female with history of vulvar cancer undergoing follow-up imaging. Coronal and axial T2WI of the pelvis demonstrates an intermediate or hypointense midline vulvar lesion (**a**, **b**) with enhancement on sagittal post-contrast imaging (**c**). While concern was initially raised that this represented recurrent disease, this was confirmed to represent a normal clitoris following further review and physical examination. Comparison with prior imaging performed 7 years earlier (**d**) confirmed similar size and configuration of the clitoris

**Fig. 7:**
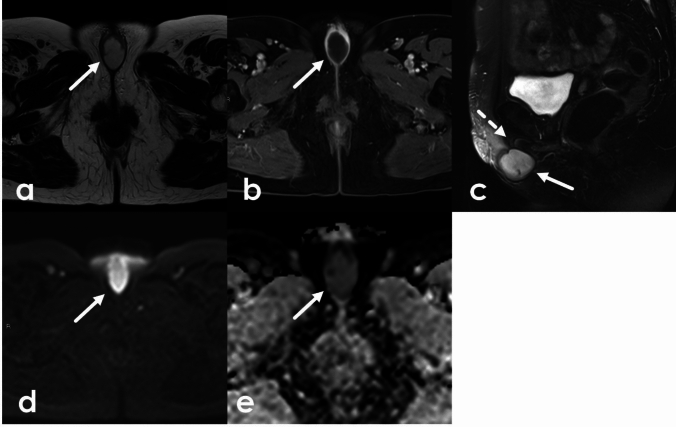
68-Year-old female with clitoral enlargement. Axial (**a**) and sagittal T2WI (**c**) demonstrates a T2 hyperintense lesion (white arrow) located inferior and slightly anterior to the glans of the clitoris (dashed white arrow). Post-contrast T1WI demonstrates rim-enhancement (**b**), and DWI/ADC demonstrates diffusion restriction of this structure (**d**, **e**) consistent with clitoral hood abscess

The clitoris can vary widely in size ([1] (Fig. 6). Enlargement of the clitoris, known as clitoromegaly, can be caused by both congenital causes, such as in CAH, or acquired causes, such as medications used in preparation for metoidioplasty. Additional conditions which cause an apparent enlargement of the clitoris include clitoral abscesses or epidermoid cysts. Vulvar abscesses appear similarly to soft tissue abscesses elsewhere, with variable T1 and T2 signal, rim enhancement on postcontrast imaging and diffusion restriction on DWI/ADC (Fig. 7). Inflammatory changes in the tissues can appear as mass-like vulvar enlargement on physical examination.

## Imaging and pathology of the labia

### Vascular pathologies

**Fig. 8:**
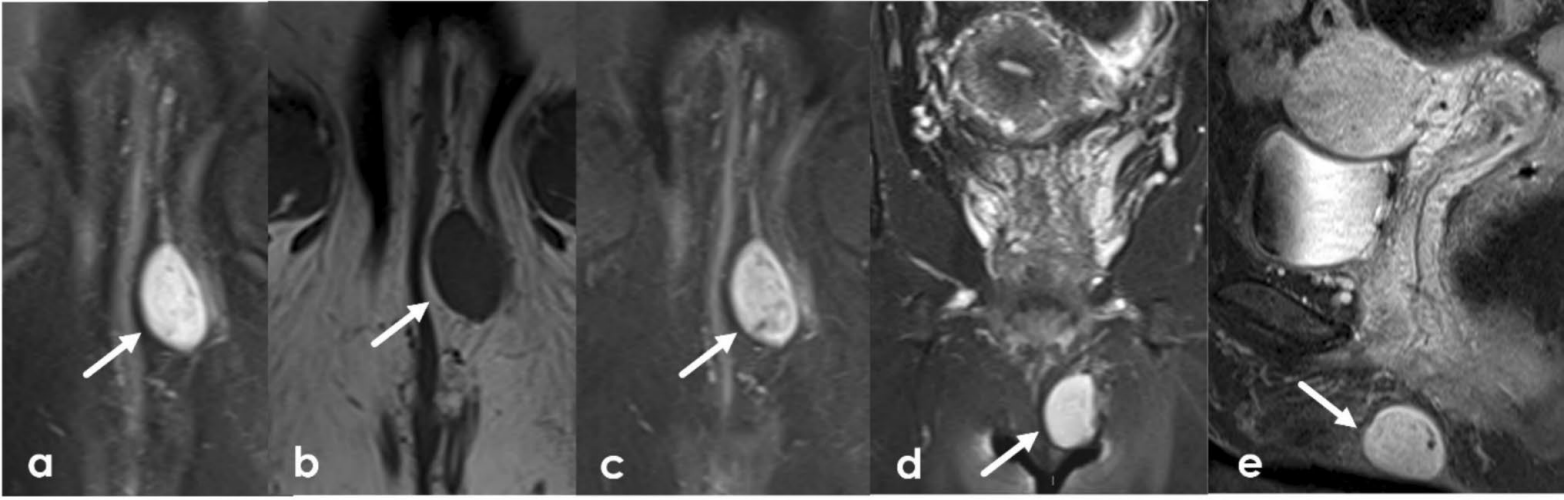
49-Year-old female with a painful left vulvar mass. T2FS axial and coronal imaging of the pelvis demonstrates an ovoid T2 hyperintense (**a**, **d**) lesion centered in the left labia that is T1 hypointense on axial T1 pre-contrast images (**b**) and demonstrates enhancement on post contrast imaging (**c**, **e**). Given multiple unsuccessful attempts at in-office resection, the patient underwent radical left vulvectomy. Biopsy confirmed a diagnosis of sinusoidal variant vulvar hemangioma

**Fig. 9:**
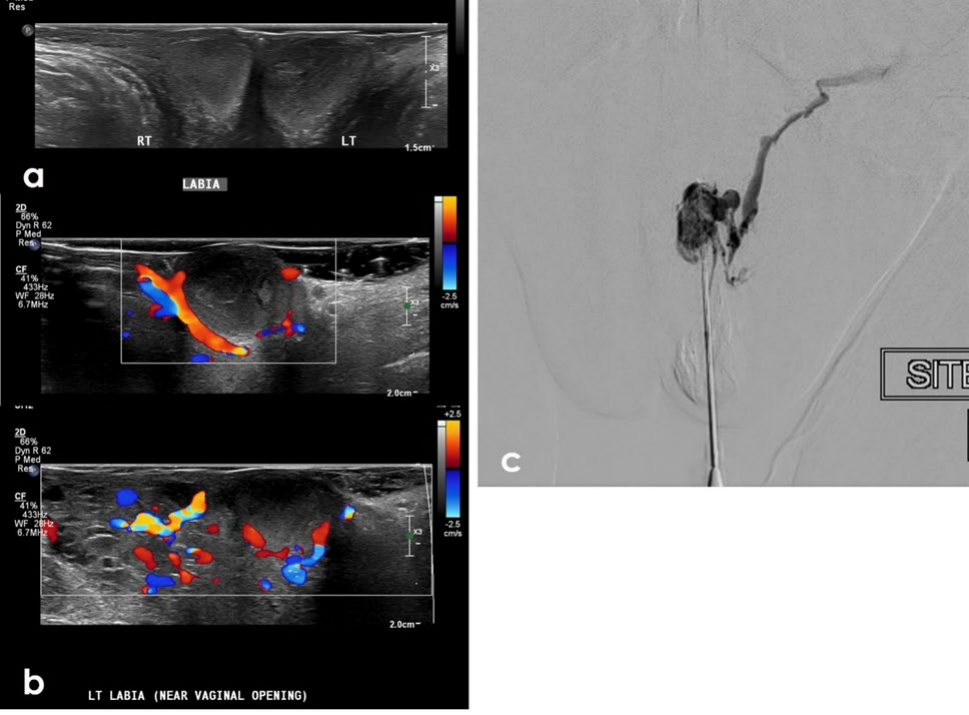
71-Year-old female with palpable labial mass. Grayscale (**a**) and color ultrasound (US) (**b**) images of the external genitalia show hypoechoic labial masses bilaterally. Tortuous vessels were noted in the adjacent soft tissues as well as the periphery of the lesions, which demonstrated slow flow internally on cine imaging (not shown). A diagnosis of labial varices was suggested. Embolization was performed by interventional radiology with resolution of the lesions and symptoms (**c**)

Vascular pathologies can affect the vulva and will be similar in appearance as at other locations. These include venous malformations, arteriovenous malformations (AVMs), hemangiomas, and varicose veins (Fig. 8). The most affected site is the perineum, particularly the labia majora [25] (Fig. 9). Vascular pathologies can be limited to the vulva or appear in conjunction with malformations elsewhere, particularly in the setting of underlying syndromes. Arteriovenous malformations, venous malformations and hemangiomas are rare and are more often related to diffuse conditions affecting the lower limbs; these include Klippel-Trenaunay syndrome and Bockenheimer syndrome [26]. Varicose veins of the vulva manifesting as labial varices are common and can occur in any condition related to pelvic outflow issues or venous insufficiency including compression from pelvic tumors or ovarian vein incompetence [26]. They are particularly common in pregnancy both due to impaired pelvic venous outflow related to the gravid uterus and effects of progesterone on the veins themselves. Around 10% of pregnant patients will develop vulvar varicose veins, which may persist after delivery [26]. Vascular lesions of the vulva can usually be evaluated clinically with limited role for imaging. However, ultrasound and MRI can be helpful for determining extent, surgical planning, and characterization when there is diagnostic uncertainty.

### Cutaneous conditions of the labia

Various skin conditions may involve the vulva. These include folliculitis, benign and malignant skin tumors, and other cutaneous pathologies. Imaging has a limited role in the diagnosis and characterization. The most common malignant skin cancers that affect the vulva, including the labia, are squamous cell carcinoma (SCC) and melanoma.

## Mesenchymal lesions of the vulva

**Fig. 10:**
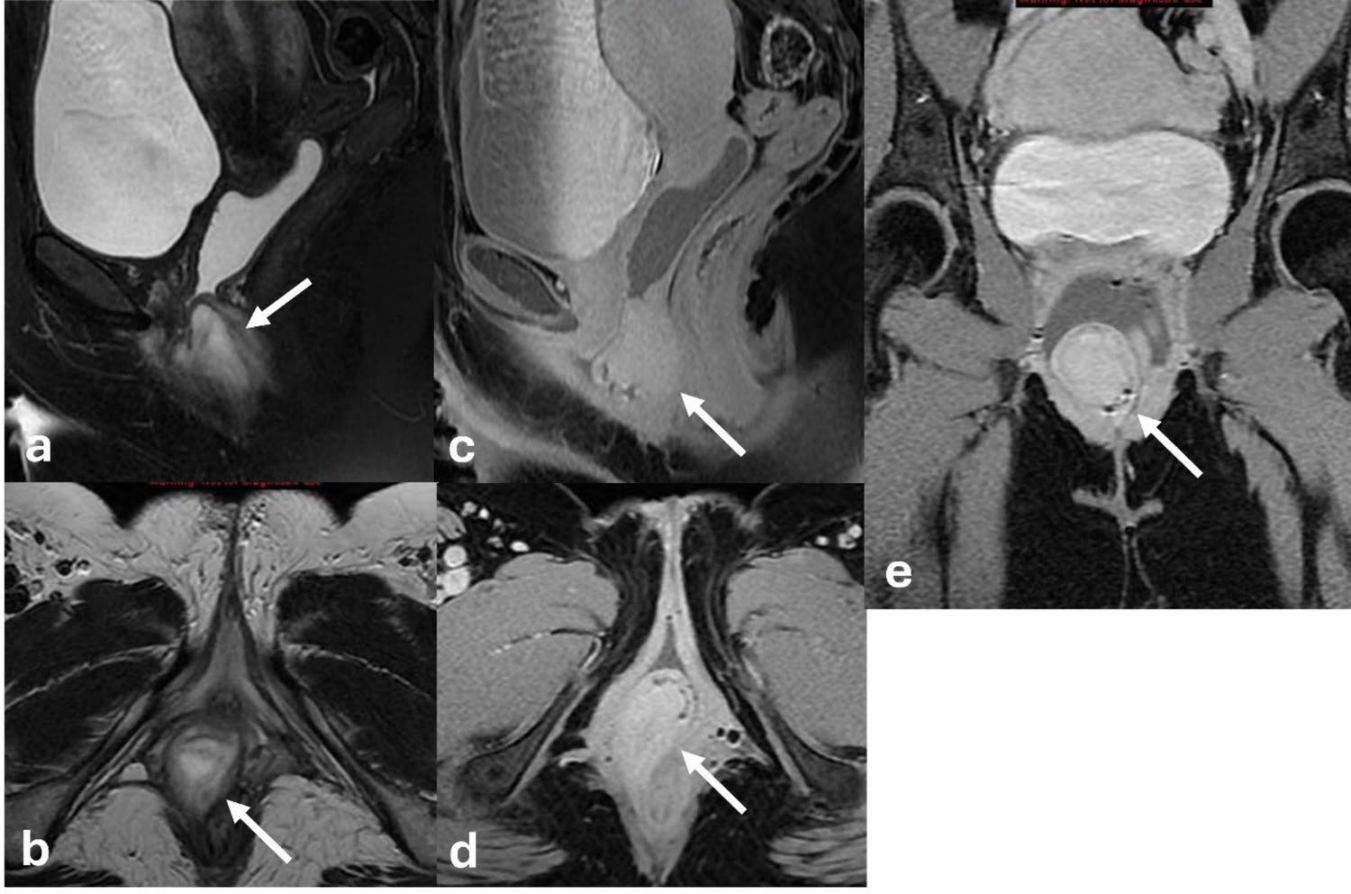
43-Year-old female presenting without significant past medical history presenting with vaginal discomfort without associated bleeding or discharge. A right posterior vaginal mass was discovered on physical exam. MRI was obtained for further characterization. This individual underwent subsequent surgical resection with pathology confirming aggressive angiomyxoma. Sagittal fat-saturated T2WI and axial non fat-saturated T2WI of the pelvis following administration of vaginal gel demonstrate a T2 heterogeneous lesion (arrow) centered in the right lateral aspect of the vagina extending to the introitus. The lesion demonstrates uniform enhancement on sagittal (**a**), axial (**d**) and coronal (**e**) post-contrast T1WI

**Fig. 11:**
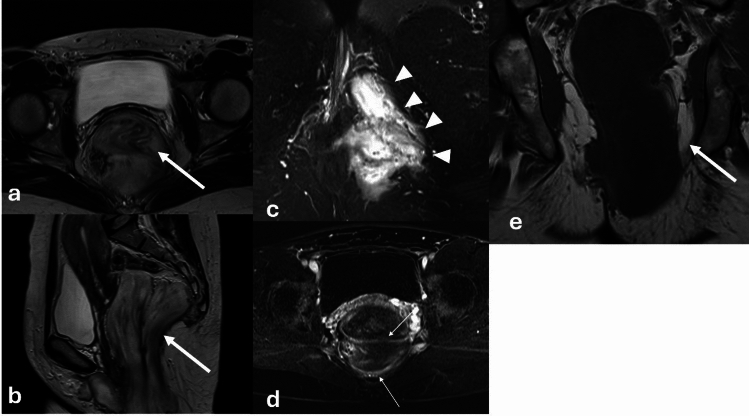
51-Year-old female without significant past medical history presenting with approximately 1 year of pelvic and perineal fullness, more apparent during exercise. Two months prior to presentation, the patient noticed a bulge in the left vulvar region and new onset constipation which prompted an OB/GYN appointment. A palpable mass was confirmed on physical exam and a pelvic MRI was obtained which showed an approximately 15 cm bilobed mass in the low pelvis between the rectum and vagina extending through the urogenital diaphragm and into the left perineum. The mass demonstrated homogeneous low signal on T1, high signal on T2 with swirled low intensity bands which enhanced on post contrast imaging. The patient was treated preoperatively with GnRH analog injections in an effort to shrink the mass followed by surgical debulking. Pathology confirmed aggressive angiomyxoma. Axial (**a**) and sagittal (**b**) T2-weighted images of the pelvis demonstrate a T2 hyperintense lesion (arrow) with swirling T2 hypointense internal striations centered in the rectovaginal space, traversing the urogenital diaphragm and extending inferiorly to the left perineum. Perineal extent is best appreciated on fat-saturated axial T2-weighted images (**c**) (arrowheads). The internal striations demonstrate modest enhancement (thin arrows) on axial post-contrast T1 subtraction images (**d**). On coronal T1-weighted images (**e**), the lesion is uniformly hypointense

**Fig. 12:**
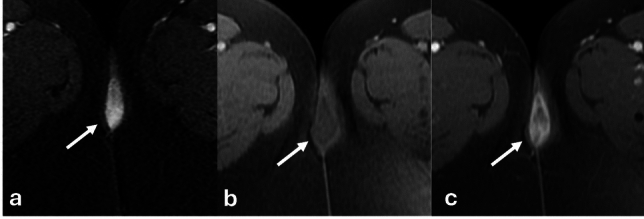
21-Year-old female with labial mass. Axial fat-saturated T2-weighted images (**a**) and pre- and post-contrast T1-weighted images (**b**-**c**) demonstrate a uniformly T2 hyperintense, heterogeneously enhancing mass (arrow) at the midline vulva. The patient underwent surgical resection, with pathology showing angiomyofibroblastoma

Mesenchymal tumors of the vulva are rare and range from benign angiomyofibroblastomas (AMF) and aggressive angiomyxomas to malignant sarcomas. Aggressive angiomyxomas are rare mesenchymal tumors. They most commonly arise in the vagina but can occur anywhere in the pelvis and perineum including the vulva. Although they are considered benign and are slow growing, they are considered locally aggressive. Aggressive angiomyxomas are highly infiltrative and tend to recur. Composed of spindle cells and blood vessels interspersed throughout myxoid tissue, they are usually large at time of diagnosis and may displace structures rather than invade adjacent tissues. Aggressive angiomyxomas are well defined and highly vascular. Due to the presence of myxoid tissue they are T1 isointense or hypointense and very T2 hyperintense with low signal bands contributing to a laminated appearance on T2WI. Avid heterogeneous enhancement is often present on postcontrast imaging due to the degree of lesional vascularity (Fig. 10, 11). Treatment is surgical, and recurrence is common [27]. Aggressive angiomyxomas frequently cross the levator ani and reporting their relationship to the pelvic floor musculature is important for treatment planning. Angiomyofibroblastomas (AMF) appear similarly to aggressive angiomyxomas, with low or intermediate T1 signal, T2 hyperintense signal related to myxoid elements, and enhancement related to their vascularity (Fig. 12). Unlike aggressive angiomyxomas, they are much smaller (< 5 cm) and do not recur. Cellular angiofibromas, also known as AMF-like tumors, can appear and behave similarly to AMF. They are well circumscribed and are composed mainly of bland spindle cells. Their appearance varies on MRI and definitive diagnosis is determined by histopathology [28]. Both AMF and cellular angiofibromas can be misdiagnosed as Bartholin gland cysts, but both enhance on post contrast imaging. Vulvar sarcomas are rare, accounting for 1–2% of vulvar malignancies. The most common sarcoma is leiomyosarcoma. Their staging an appearance are further discussed in the vulvar cancer section [27].

## Vulvar cancer

Vulvar cancer is rare but comprises 4% of gynecologic cancers [29]. Most vulvar cancers are squamous cell carcinomas (SCC), accounting for approximately 90% of vulvar malignancies [30]. Malignant melanoma is the next most common, accounting for 5% of malignancies [31]. Other less common malignancies include sarcomas, Paget’s disease of the vulva and Bartholin gland adenocarcinomas [29]. The vulva can also be secondarily invaded by adjacent malignancies (Supplemental Fig. 2).

The 2021 update to the Internal Federation of Gynecology and Obstetrics (FIGO) staging of vulvar cancer utilizes the same staging schema for all vulvar cancers, except melanoma. Previously, the schema only applied to SCC. Given the rarity of vulvar cancers that are neither SCC nor melanoma, this review will focus on SCC and melanoma and only briefly discuss the less common malignancies, with the understanding that the same FIGO 2021 staging paradigm as SCC is applied.

### Epidemiology and presentation of squamous cell carcinoma

Vulvar SCC typically affects patients greater than 60 years of age, the incidence has been increasing in younger patients, usually secondary to human papilloma virus (HPV) infection and HPV-related oncogenesis [32]. HPV-related vulvar SCCs are associated with infection with high-risk strains of the virus including subtypes 16 and 18 [32–34]. Precursor lesions are called vulvar intraepithelial neoplasia (VIN) [34]. Non-HPV related vulvar SCCs often affect older patients. These typically arise in clinical situations associated with chronic inflammation such as lichen sclerosis [35]. Patients may be asymptomatic or present with bleeding, pruritis and pain. These lesions are usually visible on clinical exam and can appear as discolored, thickened ulcerations or plaque-like areas on the skin [36].

### Pathways of spread

Vulvar cancers usually spread by direct extension to adjacent structures including the vagina, urethra, and anus as well as through the lymphatics. The superficial inguinal lymph nodes are most commonly involved, but the deep inguinal and external iliac lymph nodes may be involved as well, including spread to the deep inguinal lymph nodes without involvement of the superficial lymph nodes. Lesions that involve the lateral vulva typically spread to the ipsilateral inguinal lymph nodes. Contralateral lymph node involvement is extremely rare in the absence of ipsilateral inguinal lymph node involvement in these cases [37]. Midline structures, or those very close to midline, can drain to either or both sides due to the significant anastomotic network in this region. The inguinal lymph nodes drain into the external iliac chain. Involvement of pelvic lymph nodes is not regional and considered metastatic [36].

### Imaging and staging

**Fig. 13:**
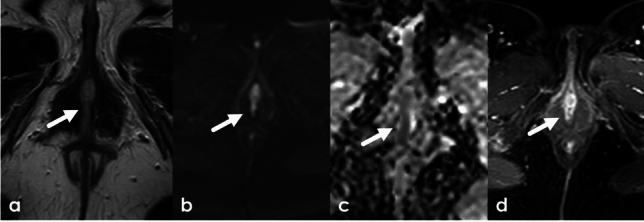
64-Year-old female with visible ulcerated erosive vulvar lesion. Axial T2WI demonstrates a T2 intermediate midline vulvar mass (**a**) with diffusion restriction (**b**, **c**) on DWI/ADC, and enhancement on axial and post-contrast images (**d**). Biopsy confirmed a diagnosis of squamous cell carcinoma

**Fig. 14:**
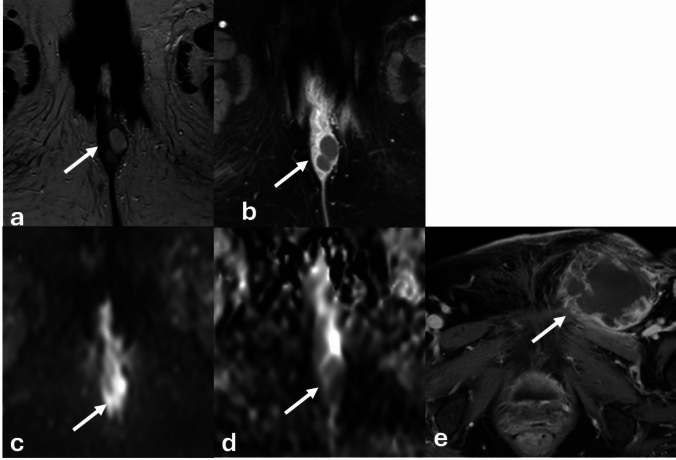
51-Year-old female with vulvar and left groin masses. Axial non-fat saturated (**a**), T1 post-contrast (**b**, **e**) and diffusion weighted MR images of the pelvis with accompanying ADC map (**c**-**d**) demonstrate an ovoid, uniformly enhancing T2 hypointense vulvar mass (arrow) with central cystic component. The lesion demonstrates diffusion restriction. Bulky, necrotic metastatic adenopathy is noted in the left groin (arrowhead)

Generally, lesions are biopsied prior to imaging. The role of imaging is therefore largely for assessing local extent and staging. Vulvar cancer will demonstrate intermediate T2 signal and variable enhancement on postcontrast imaging with associated diffusion restriction (Fig. 13, 14). In patients who have undergone treatment, residual or recurrent tumor will demonstrate intermediate T2 signal and early enhancement in contrast to radiation fibrosis which will be T2 hypointense and demonstrate late enhancement on DCE. The 2021 update for FIGO staging of vulvar cancer also includes the incorporation of cross-sectional imaging in staging and is detailed in Table 3 [29].

**Table 3:** Staging vulvar cancer

Stage 1 *Disease confined to the vulva*	IA: 1mm or less of stromal invasion *and* 2 cm or below in size IB: Either > 1mm of stromal invasion *and/or* > 2cm in size
Stage 2 *Locally invasive disease*	Any sized tumors that invade the lower one third of the urethra, vagina or anus
Stage 3 *Any nodal involvement automatically stage 3 or above*	IIIA: Local invasion of any structure not described in stage II, including the upper 2/3 of the vagina, the bladder or rectum *and/or* lymph nodes 5 mm or less in size IIIB: Lymph nodes > 5 mm in size IIIC: Lymph nodes with ECS
Stage 4	IVA: Tumor is fixed to bone or ulcerated lymph nodes IVB: Distant metastases

*ECS* extracapsular spread

### Reporting

When interpreting cross-sectional imaging of the pelvis for a patient with vulvar cancer, key features should be described including: the size of the primary tumor in multiple planes and the location (including whether the tumor is midline or close to midline), and specific structures involved by tumor spread, including the clitoris, urethra, bladder, vagina, posterior fourchette, perineal skin, anus, or rectum. Lymph node descriptions should include the number of suspicious lymph nodes present as well as their size, location, and whether there is suspicion for extracapsular spread or ulceration. Additional findings to report include osseous involvement and presence of distant metastatic disease.

### Vulvar melanoma

**Fig. 15:**
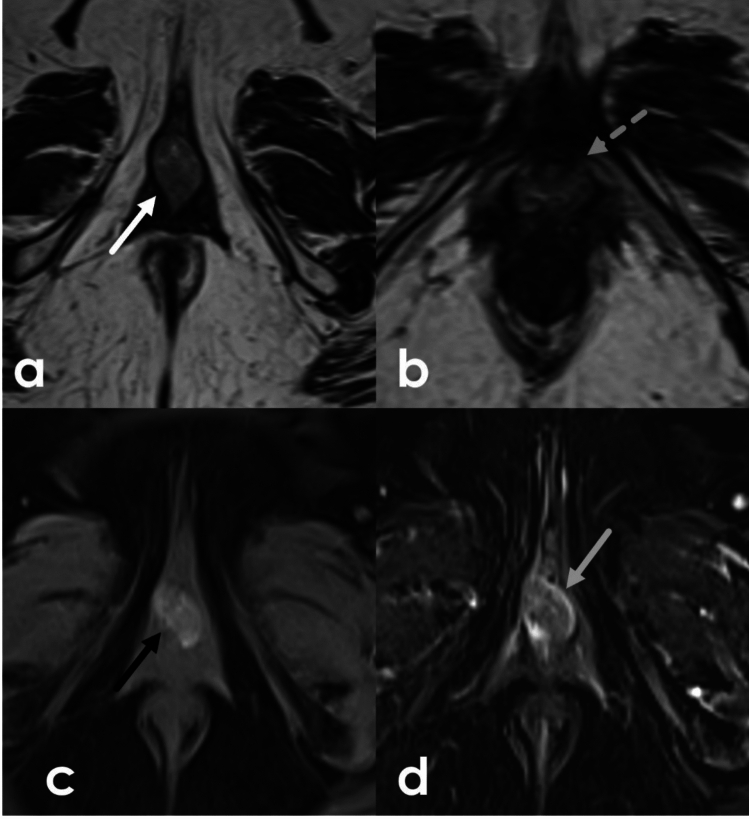
64-Year-old female with vaginal and vulvar melanoma. Axial 3D T2-SPACE (**a**, **b**) images demonstrate a mass centered in the vaginal introitus (white arrow) and encircling the urethra (dashed gray arrow). Axial T1FS pre-contrast (**c**) demonstrates intrinsic T1 signal in the lesion (black arrow) and on subtraction post-contrast T1FS (**d**) demonstrates enhancement (grey solid arrow)

Vulvar melanomas are the second most common vulvar malignancy and refer to a subset of melanomas called mucosal melanomas which affect the labia minora, the clitoris, the inner labia majora, and the vestibule. Melanomas of the mons pubis and outer labia majora are treated as cutaneous melanomas elsewhere in the body. Vulvar melanomas most commonly involve the clitoris and the labia minora [29]. Patients often present with pigmented, or in some cases amelanotic or red lesions on physical exam, which may cause irritation, pruritis, pain or bleeding [38]. Vulvar melanomas will contain a variable amount of melanin. Lesions with high melanin content will demonstrate high T1 signal due to the paramagnetic effects of melanin. In tumors with low melanotic content or completely amelanotic tumors, tumors may appear low or intermediate in signal intensity on T1 weighted imaging. The incidence of macroscopically amelanotic tumors has been described as approximately 30% of vulvar melanomas. However, even macroscopically amelanotic tumors can contain microscopic amounts of pigment, resulting in variable appearance on MRI [39]. Vulvar melanomas enhance on postcontrast imaging with associated diffusion restriction (Fig. 15). Necrosis may appear as foci of hypoenhancement. There is no consensus on the staging of mucosal melanomas, including vulvar melanomas, though the American Joint Committee on Cancer (AJCC) system is favored over the FIGO classification used for other vulvar cancers as the AJCC system incorporates Clark and Breslow classifications of tumor depth of invasion and size; these schemas have been shown to correlate with survival. Like vulvar SCC, vulvar melanoma is treated with wide local excision. Any suspicious lymph nodes on imaging should be removed, but otherwise the role of lymph node dissections in melanoma is less well defined compared to SCC [29].

### Vulvar sarcomas

Vulvar sarcomas are rare and account for 1–2% of vulvar malignancies. The most common vulvar sarcomas are leiomyosarcomas, but other entities including rhabdomyosarcoma and malignant fibrous histiocytoma can occur as well. Vulvar sarcomas most commonly occur in the labia but can occur elsewhere. Sarcomas in the Bartholin gland region can be mistaken for Bartholin gland cysts and abscesses, so care should be taken to avoid this pitfall. Any growing mass should raise concern, especially if no inflammatory findings suggestive of abscess are observed. Clinically, vulvar sarcomas can be painful, pruritic, or demonstrate ulceration or bleeding. On imaging, they appear similarly to SCC though likely less infiltrative compared to SCC [27]. They are heterogeneous on T2WI, with hyperintense areas if necrosis is present, and hypointense or intermediate in signal on T1WI. Like other malignancies, they restrict diffusion and demonstrate heterogeneous enhancement [27]. There may be foci of nonenhancement if necrosis is present. Given their rarity, there are limited guidelines for the treatment of vulvar sarcoma but they are often treated with wide local excision similarly to vulvar SCC [40].

## Bartholin glands

### Bartholin gland cysts

**Fig. 16:**
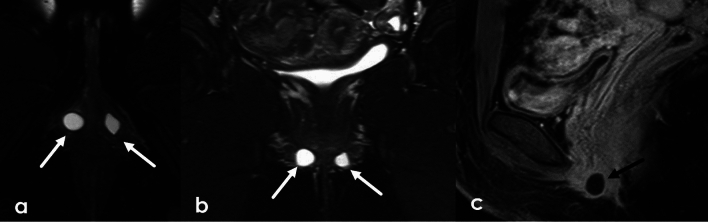
34-Year-old female undergoing MRI for uterine fibroids with incidentally noted Bartholin gland cysts. Axial and coronal fat saturated T2WI demonstrates bilateral cystic lesions located posterior and lateral to the vaginal introitus (white arrows). Sagittal contrast enhanced T1WI demonstrates nonenhancement of a cyst (black arrow). Note location below the pubic symphysis

**Fig. 17:**
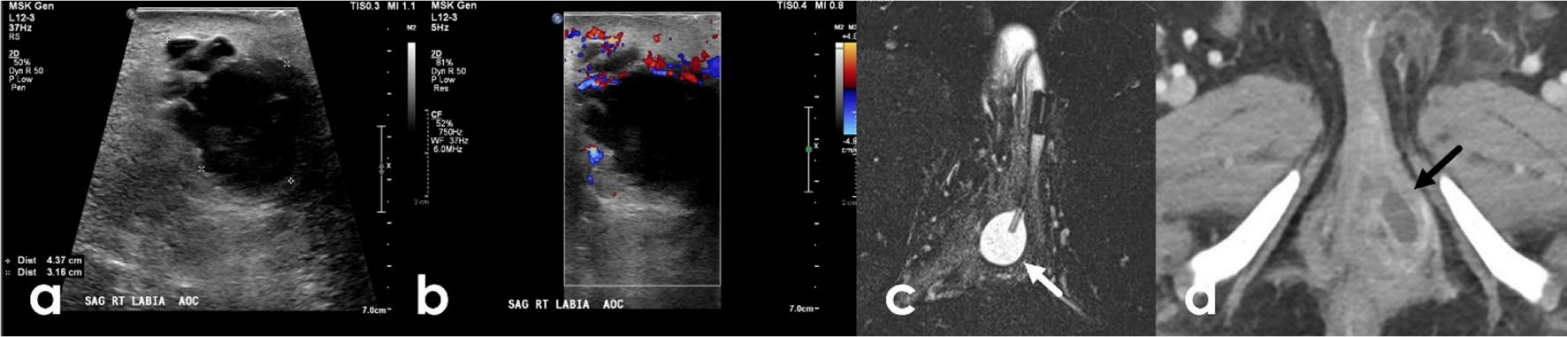
**a**–**c**. 26-year-old female with Bartholin gland abscess. Greyscale and color US images of the region of the Bartholin gland demonstrate a complex collection with surrounding hyperemia. Axial high res T2FS demonstrates a Bartholin gland abscess after placement of Word catheter (white arrow). **d** 85-year-old female with Bartholin gland abscess. Axial contrast enhanced CT images demonstrate a large rim enhancing collection in the area of the Bartholin gland with surrounding tissue stranding (black arrow)

Bartholin gland cysts are often unilocular, located on either side of the posterior vagina, medial to the labia minora, at or below the level of the pubic symphysis (Fig. 16). They are usually unilateral, but they can be bilateral. As fluid containing structures, they measure fluid density on CT and have high T2 signal on MR. They can have variable T1 signal and be more dense on CT particularly in the setting of inflammation or infection. These ducts can become obstructed which can result in superinfection (Fig. 17).

### Bartholin gland neoplasms

Rarely, neoplasms can arise in the Bartholin glands. Bartholin gland cancers have varying appearances. Nearly all patients present with a mass in the region of the Bartholin gland. Squamous cell carcinomas arise from the orifice of the duct, where it empties into the vagina, and adenocarcinomas arise from the remainder of the duct, in keeping with the glandular histology. These malignancies are staged and treated similarly to other vulvar cancers with the goal of therapy focused on radical resection of the primary lesion and ipsilateral inguinal lymphadenectomy [29]. Their location can pose challenges for adequate margins, necessitating radiation in many patients [37]. Vulvar soft tissue neoplasms including benign entities such as angiomyofibroblastoma, primary vulvar sarcomas, and cancer spread from elsewhere can have overlapping features with Bartholin gland cysts or abscesses [27].

## The urethra and skene’s glands

### Imaging appearance of the urethra

The outermost layer of the urethra is composed of striated muscle and is T2 hypointense. The inner layers are composed of smooth muscle and submucosa which are both hyperintense on T2. The innermost layer, the mucosa, is T2 hypointense [41] (Fig. 3).

### Urethral diverticula

**Fig. 18:**
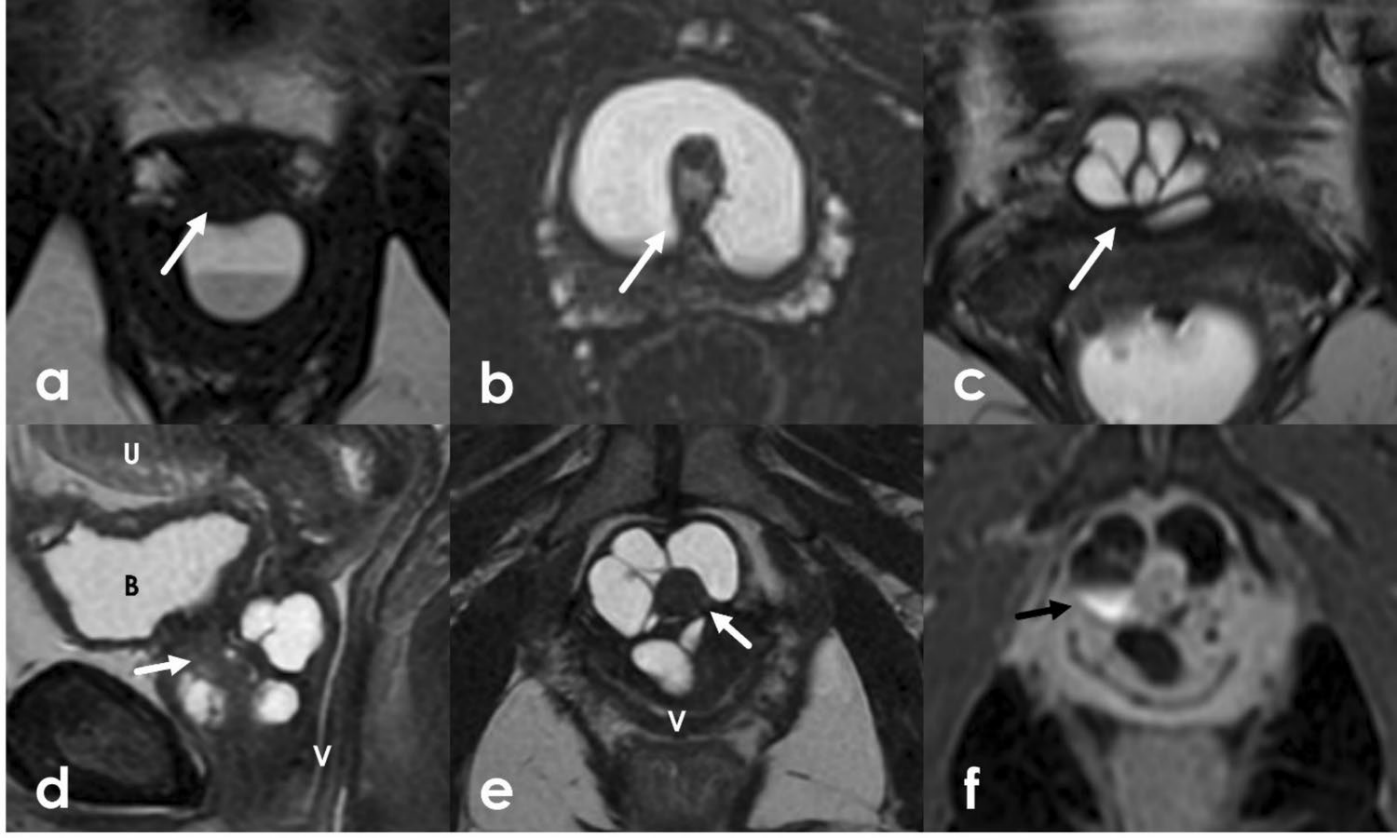
**a**–**c** axial T2 high resolution imaging of the urethra in three different female patients with urethral diverticula. Simple diverticulum posterior to the urethra (arrow) (**a**) with layering debris. U-shaped diverticulum encircles the urethra (arrow) (**b**). Complex diverticulum anterior to the urethra (arrow) (**c**). **d-f.** 49-Year-old female with a history of previously excised diverticulum. Sagittal T2 (**d**), Axial oblique T2 (**e**), and post-void, contrast-enhanced axial T1FS oblique sequences (**f**) of the urethra demonstrates a complex cystic structure arising encircling the female urethra (white arrow). Postvoid contrast enhanced imaging shows filling of the cystic structure with excreted contrast (black arrow) demonstrating communication with the urethra consistent with recurrent diverticulum. *U* uterus, *B* bladder, *V* vagina

Urethral diverticula are projections of the urethra into the surrounding fascia. They are true diverticula with an epithelial lining identical to the urethral mucosa. Symptoms are nonspecific but patients may present with the classic triad of dysuria, dyspareunia, and postvoid dribbling. They appear hyperintense on T2 weighted imaging but can have varying signal if they contain proteinaceous or hemorrhagic contents. They can be single, multiple, short or wide-necked, and can demonstrate various degrees of extension around the urethra. Possible configurations range from simple oval or round shapes, partially circumferential (U-shaped), or completely circumferential, called “saddlebag” diverticula (Fig. 18). They do not enhance but can retain contrast on postvoid imaging as excreted contrast fills the bladder and the urethra. Diverticula can become superinfected or develop calculi or neoplasms within the diverticula themselves. Malignancy within diverticula is extremely rare. The most common neoplasm is clear cell adenocarcinoma [42].

### Urethral cancer

Urethral cancer is rare. Risk factors for urethral cancer include chronic urinary tract infections, human papillomavirus infection (HPV), conditions such as leukoplakia and urethral diverticula. The most common urethral malignancy is squamous cell carcinoma (SCC) in the distal two thirds of the urethra, which is lined by squamous epithelium. The proximal third of the urethra may develop urothelial carcinoma, reflecting the urothelial cells which line the mucosa [41]. The urethra can also be involved by vaginal or vulvar tumors. Urethral malignancies involving only the distal third of the urethra are called anterior urethral tumors. The remainder of urethral tumors are called entire urethral tumors [43]. This distinction is important as anterior urethral tumors have a better prognosis, different lymphatic drainage and are managed differently than entire urethral tumors. Anterior urethral malignancies drain via the inguinal lymph nodes and are treated with local excision. Entire urethral tumors drain via the external iliac, hypogastric, obturator, and paraaortic lymph nodes. They often require a more extensive combination of therapies including resection and chemoradiation [43].

### Urethral malignancy imaging appearance

**Fig. 19:**
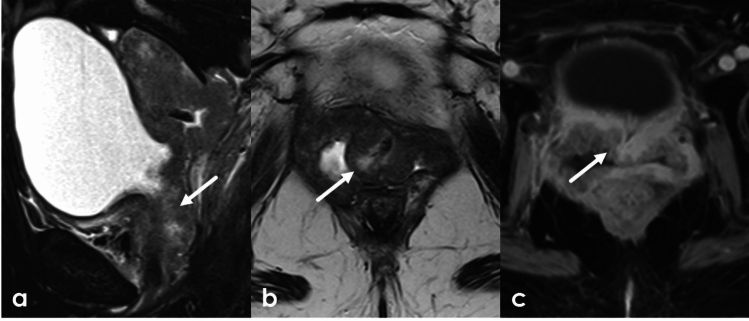
52-Year-old female with history of cervical cancer status post chemoradiation with vaginal pain and bleeding. Sagittal and axial T2WI of the pelvis demonstrates mass-like thickening of the cervix and vagina with enhancement and circumferential involvement of the urethra (arrows). Pathology revealed recurrent poorly differentiated invasive squamous cell carcinoma

Urethral tumors are best appreciated on sagittal and axial imaging of the urethra. Lesions are most often T1 hypointense, T2 intermediate, enhance on post contrast imaging, and restrict diffusion (Fig. 19) [43].

### Skene’s gland cysts

**Fig. 20:**
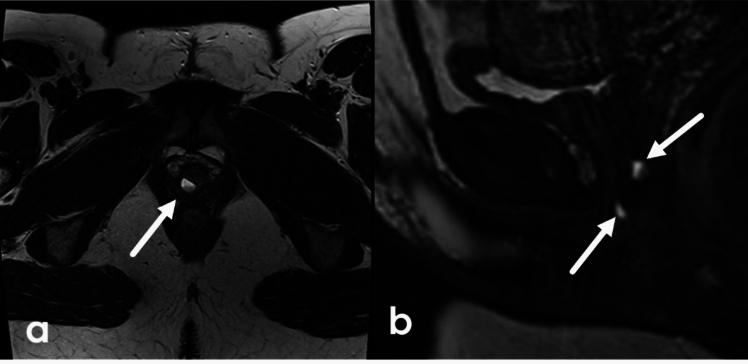
37-Year-old female undergoing pelvic MRI for abnormal uterine bleeding with incidentally noted periurethral cyst. Skene’s gland cysts are often located lateral to the urethra. This may help in distinguishing them from urethral cysts which may be more posterior in location

The Skene’s (paraurethral) glands are paired glands that open into the distal urethra. The glands are composed of mucinous columnar epithelium and their ducts are lined by columnar epithelium [7]. Benign cysts can occur within the ducts (Fig. 20).

## Conclusion

Historic disparities in the study of women’s health have resulted in a paucity of information on the topic of vulvar anatomy and pathology, especially compared to information regarding male genitalia or compared to other areas of gynecology and obstetrics such as pregnancy and childbirth. This disparity can contribute to poor patient outcomes as vulvar pathologies can greatly impact an individual’s quality of life or life expectancy.

Radiologists must have an understanding of vulvar anatomy to ensure accurate interpretation of vulvar imaging as well as familiarity with the numerous varied conditions that can affect the vulva, including congenital and acquired conditions and benign and malignant pathologies (Table 4). Accurate identification and characterization of these conditions can improve patient care by aiding in surgical planning, preventing unnecessary disfiguring or life altering surgeries, detecting cancer recurrences early, and accurately describing injuries related to perineal trauma.

**Table 4:** Benign and malignant masses or mass-like lesions of the vulva

Pathology	Location	Benign/malignant	Imaging appearance
Abscess	Varies	Benign	Variable on T1W and T2W. Restricts diffusion. Rim enhancement or heterogeneous enhancement
Cyst	Varies (Bartholin gland, Skene’s gland, Gartner duct)	Benign	If simple, hypointense on T1W and hyperintense on T2WI, but can vary depending on cyst contents or if superinfected
Diverticula	Urethra	Benign; (rarely, carcinomas can develop within a diverticulum)	T2 hyperintense, T1 hypointense, but can vary depending on contents. Does not enhance. Can retain excreted contrast on post void imaging. Demonstrates various degrees of extension around the urethra with many possible configurations
Varices	Varies	Benign	T2 hyperintense and enhance on post contrast imaging
Hemangioma	Varies	Benign	T2 hyperintense, T1 hypointense, avid enhancement
Angiomyofibroblastoma	Superficial soft tissues	Benign	Small well circumscribed mass (< 5cm). Can be mistaken for a Bartholin gland cyst Similar in appearance to AA (though smaller)
Cellular Angiofibroma	Superficial soft tissues	Benign	Small well circumscribed mass (< 5 cm). Variable signal. Can be mistaken for a Bartholin gland cyst
Aggressive Angiomyxoma	Vagina (usually); can involve the vulva	Benign (Locally aggressive)	T1 isointense or hypointense, T2 hyperintense with interspersed low signal bands contributing to a “laminated appearance” on T2W. Avid heterogeneous enhancement
Carcinoma (most commonly SCC)	Varies	Malignant	Intermediate on T2; hypointense on T1 Restricts diffusion and demonstrates enhancement on postcontrast imaging
Melanoma	Varies	Malignant	Intermediate T2; variable T1(can be very hyperintense on T1 if pigmented). Restricts diffusion and demonstrates enhancement on post-contrast imaging
Sarcomas (most commonly LMS)	Varies (Labia most commonly; can be mistaken for a Bartholin gland cyst or abscess if located in the region of the Bartholin gland)	Malignant	Heterogeneous on T2W, with hyperintense areas if necrosis is present. Restricts diffusion and demonstrates heterogeneous enhancement on post-contrast imaging

*AA* aggressive angiomyxoma, *SCC* squamous cell carcinoma, *LMS* leiomyosarcoma, *T1W* T1-weighted imaging, *T2W* T2-weighted imaging

Radiologists can also have an outsized impact in the care of these patients beyond the workstation. Radiologists are needed for better optimization and standardization of vulvar imaging protocols as well as their dissemination with the potential to positively impact female patients worldwide. Additionally, as a result of medical advances and increased globalization, radiologists will encounter altered vulvar anatomy in novel patient care settings, such as in the case of providing gender affirming care and in the care of patients with a history of female genital mutilation. In collaboration with anatomists, surgeons, urologists, and gynecologists, radiologists have the opportunity to contribute to further advances in scholarship in this field and address the knowledge gaps that have negatively impacted female sexual health for far too long.

## Supplementary Information

Below is the link to the electronic supplementary material.

Supplementary file1 (DOCX 345 KB)

## Data Availability

No datasets were generated or analysed during the current study.
