# Supplementary material for: Under the hood: vulvar anatomy and pathology with a focus on MRI
Source: Abdom Radiol (NY). 2025 Sep 8;51(4):2106–24. doi: 10.1007/s00261-025-05179-1 (PMC13013239; doi:10.1007/s00261-025-05179-1)
Supplement: Supplementary file 1 — Supplementary file1 (DOCX 345 KB) [file 261_2025_5179_MOESM1_ESM.docx]

Supplemental Figures


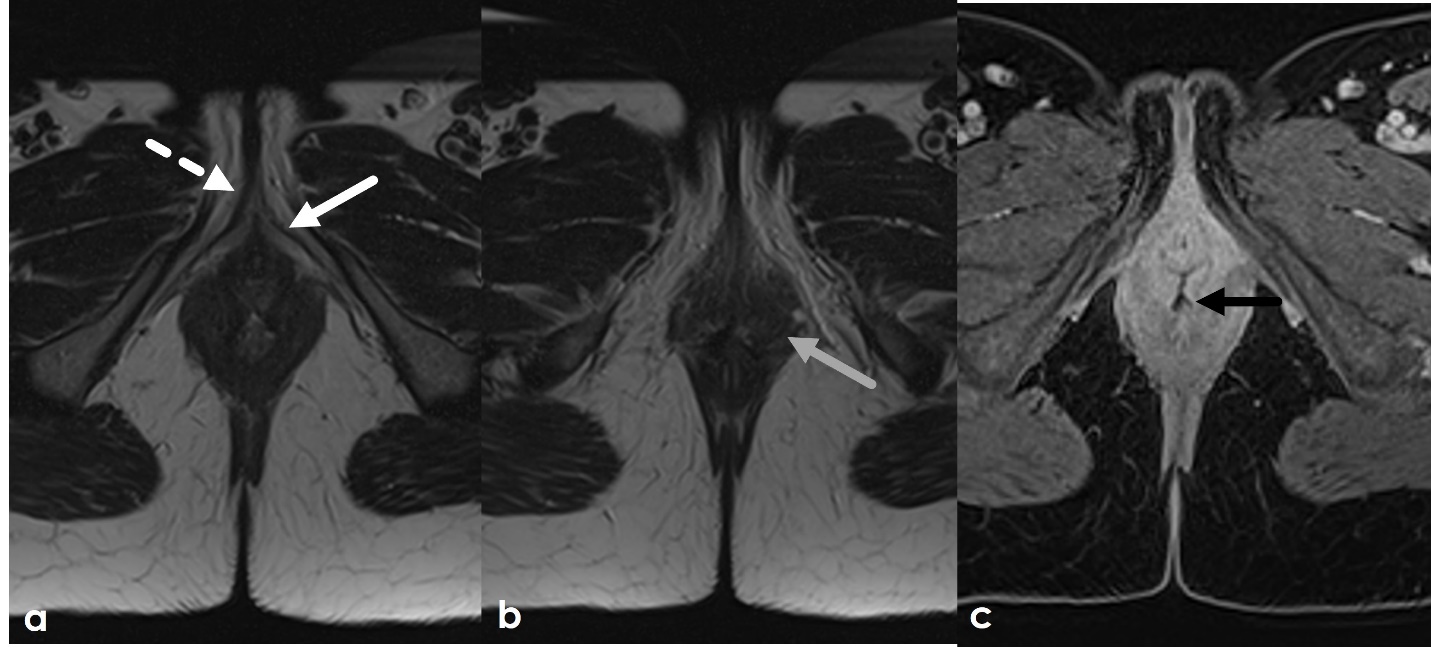

**Supplemental Figure 1.** 28-year-old female with a history of FGM as a child. Axial T2WI demonstrates a truncated appearance of the clitoris with only the crura present (white arrow) and no glans (white dashed arrow) and scarring of the labia and other vulvar soft tissues (gray arrow). Postcontrast T1WI with fat saturation demonstrates a small defect in the anus extending to vagina (black arrow), likely also secondary to genital mutilation.


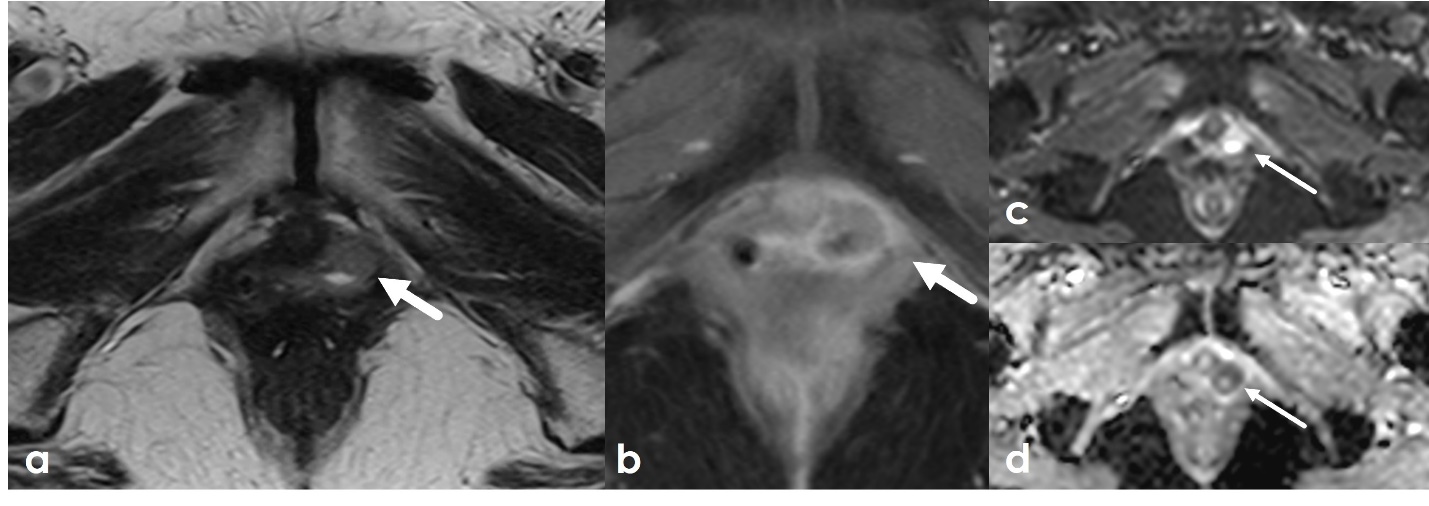


**Supplemental Figure 2**. 68-year-old female with history of stage IIIC grade 3 endometrial cancer undergoing evaluation for suspected 'vaginal/vulvar cyst' based on physical exam. Axial T2WI (a), post-contrast T1 (b), and DWI and ADC (c-d) MR images of the pelvis demonstrate a T2 intermediate lesion centered in the vaginal wall with central T2 hyperintensity, with enhancement and associated diffusion restriction. Biopsy confirmed recurrent endometrial cancer.
